# Assessment of memory complainers in São Paulo, Brazil: Three-year results of a memory clinic

**DOI:** 10.1590/S1980-57642009DN20100011

**Published:** 2008

**Authors:** Cássio Machado de Campos Bottino, Pedro Zucollo, Maria Del Pilar Quintero Moreno, Gislaine Gil, Carla Garcia Cid, Eduardo Vilodres Campanha, Demétrio Ortega Rumi, Cândida Helena Pires de Camargo

**Affiliations:** 1 MD, PhD, Psychiatrist, PROTER Coordinator. PROTER (Old Age Research Group), Institute and Department of Psychiatry - School of Medicine, University of São Paulo.; 2 Neuropsychologist. PROTER (Old Age Research Group), Institute and Department of Psychiatry - School of Medicine, University of São Paulo.; 3 MD. PROTER (Old Age Research Group), Institute and Department of Psychiatry - School of Medicine, University of São Paulo.; 4 MD, PhD, Psychiatrist. PROTER (Old Age Research Group), Institute and Department of Psychiatry - School of Medicine, University of São Paulo.

**Keywords:** memory clinic, Alzheimer’s disease, cognitive disorders, depression, clínica de memória, doença de Alzheimer, transtornos cognitivos, depressão

## Abstract

Memory clinics were established in the USA and European countries as services to attend patients who complain of cognitive deficits, referred by primary care doctors, specialists and other hospitals.

**Objective:**

We aimed to describe the clinical and cognitive profile of consecutively evaluated subjects during the initial three years of service of a memory clinic from a university hospital.

**Methods:**

Subjects were submitted to a clinical work-up for dementia, which included laboratory exams, cranial computerized tomography, cognitive tests, and a comprehensive neuropsychological battery. Diagnosis was made according to ICD-10 criteria.

**Results:**

We evaluated 104 subjects (67.3% females and 32.7% males), with mean age of 59.1 years, 88.8% aged 50 years or above. Mean schooling was 9.9 years. Patients were classified into 10 different primary diagnostic categories, namely Depression (26.9%), Alzheimer’s disease (17.3%), Memory complaints without objective impairment (17.3%), Mild Cognitive Disorder – MCD (14.4%), and Anxiety (12.5%) the most frequent diagnosis. Comparing patients with dementia, MCD, Depression or Anxiety and Memory complainers, by age (below and above 60 years), dementia was more commonly diagnosed in older subjects, while a higher frequency of memory complainers was found in the younger group.

**Conclusions:**

This preliminary report from an outpatient group of referred patients with cognitive complaints showed a higher frequency of psychiatric disorders in this sample. The memory clinic approach should be considered as a model of service which can evaluate subjects with cognitive complaints effectively and improve the quality of care delivered to this patient group.

## Abstract (pt)

Clínicas de memória foram estabelecidas nos EUA e em países europeus como serviços para atender pacientes que se queixam de déficits cognitivos, encaminhados por médicos em cuidados primários, especialistas e outros hospitais.

**Objetivo:**

Nós pretendemos descrever o perfil clínico e cognitivo de sujeitos consecutivamente avaliados nos primeiros três anos de funcionamento de uma clínica de memória de um hospital universitário.

**Metódos:**

Os sujeitos foram submetidos a avaliação clínica para demência, incluindo exames laboratoriais, tomografia computadorizada de crânio, testes cognitivos e uma bateria neuropsicológica completa. Diagnóstico foi feito segundo critérios da CID-10.

**Resultados:**

Nós avaliamos 104 sujeitos (67,3% mulheres e 32,7% homens), com idade média de 59,1 anos, 88,8% com mais de 50 anos de idade. A escolaridade média era de 9,9 anos. Pacientes foram classificados em 10 categorias diagnósticas primárias diferentes, sendo Depressão (26,9%), doença de Alzheimer (17,3%), Queixas de memória sem comprometimento objetivo (17,3%), Transtorno cognitivo leve – TCL (14,4%), e Ansiedade (12,5%) as mais freqüentes. Comparando pacientes com demência, TCL, Depressão ou Ansiedade e aqueles com Queixa de memória, por idade (abaixo e acima de 60 anos), demência foi mais comumente diagnosticada nos idosos, enquanto maior freqüência daqueles com queixa de memória foi encontrada nos mais jovens.

**Conclusões:**

Este relato preliminar de pacientes ambulatoriais encaminhados com queixas cognitivas mostrou uma freqüência elevada de transtornos psiquiátricos nesta amostra. A abordagem da clínica de memória deveria ser considerada como um modelo de serviço para avaliar efetivamente sujeitos com queixas cognitivas, podendo melhorar a qualidade do cuidado prestado a estas pessoas.

Memory clinics are common in the USA and in European countries where services to investigate memory complaints specifically in elderly subjects have been in place since the 1980s.^1^ In the UK for example, the Maudsley Memory Clinic was set up in 1984 and its open referral system proved to be efficient in identifying patients with mild to moderate Alzheimer’s disease.^2^ In referral services of this nature, AD was frequently the most common diagnosis, but a sizeable group of subjects were classified with no psychiatric diagnosis.^1^

In Brazil, one of the first Memory Clinics was set up at the Old Age Research Group (PROTER), Institute of Psychiatry, University of São Paulo Medical School, beginning its activities in September, 1996. This service was organized to offer multidisciplinary assistance to patients older than 18 years that complain of cognitive impairment. The objectives of this new clinic were: to provide early diagnosis of AD and other types of dementia; to study subjects with mild cognitive impairment (MCI) longitudinally; to investigate risk factors for dementia; and to develop and improve instruments for the clinical diagnosis of cognitive disorders adapted to our culture.

## Objectives

In the present report, we aimed to describe the clinical and cognitive profile of the first 104 consecutively evaluated subjects during the initial three years of service of a University Hospital Memory Clinic.

## Methods

Patients were referred to the Memory Clinic by other health services (hospitals, specialty outpatient clinics, private doctors), but spontaneous demand also occurred after some initial media announcements of this new service.

An initial evaluation was performed by an experienced geriatric psychiatrist to exclude subjects with mental retardation or with chronic psychiatric disorders, without consistent memory complaints. Subsequently, a one-day evaluation was scheduled, where in the morning the patient was submitted to a comprehensive diagnostic evaluation by a resident in psychiatry, which included complete medical history, physical and neurological examination, cranial computerized tomography; workup for the differential diagnoses of dementia (complete blood count with differential; renal, liver and thyroid function tests; vitamin B12 and folic acid levels; syphilis serology; urinalysis) and the application of the Cambridge Examination for Mental Disorders (CAMDEX)^3,4^ as well as brief neuropsychological testing (CAMCOG - Cognitive Section of the CAMDEX), which included the Mini-Mental State Examination (MMSE).^5^ After lunch, a comprehensive neuropsychological battery^6,7^ was applied by a trained neuropsychologist, including the evaluation of the following cognitive functions: estimated IQ (WAIS-R: block design, vocabulary); Attention (digit span, Trail-Making A and B); Motor Functions (Luria and Ozeretski); Visuo-spatial and Motor ability (Necker Cube, watch drawing); Language (Boston Naming Test, verbal fluency); Calculation; Memory (Fuld Object Memory Evaluation, Face Recognition, Picture Recognition, Selective Reminding Test).

Syndromic diagnosis was made at the end of this first evaluation. Etiologic diagnosis was reached by consensus with at least two physicians and one neuropsychologist, and met ICD-10^8^ criteria for mental disorders 2 or 3 weeks after the first evaluation. At the time, Petersen’s criteria^9^ for MCI was still being developed and therefore the ICD-10 Mild Cognitive Disorder (MCD) term was used instead. All the subjects agreed to participate in the study.

Data analysis was performed with the *SPSS 14.0 for Windows* program. We present descriptive statistics, with frequency, percentage, mean and standard deviation. The comparison of the four most frequent syndromic diagnoses (Dementia, Depression/Anxiety, Mild Cognitive Disorder, and Memory Complainers) by age group (20 to 59 years and above 60 years) was carried out with Pearson’s Chi-square. The comparison of age and schooling of the four diagnostic groups was performed with Analysis of Variance. As the groups were significantly different regarding age and schooling, MMSE and CAMCOG score comparison among the four diagnostic groups was made with Analysis of co-variance (ANCOVA), with age and schooling as co-variates. Pairwise comparisons were corrected for multiple testing by the Bonferroni method.

## Results

One hundred and four patients were evaluated between September 1996 and April 1999. Demographic characteristics are presented in Table 1. Mean sample age was 59.1 years (SD=13.6), 88.8% aged 50 years or above. Mean schooling was 9.9 years (SD=4.8).

**Table 1:** Socio-demographic characteristics.

	n	%^a^	%^b^
**Age groups**	104		
0-29 30-39 40-49 50-59 60-69 70-79 80-89	2 9 11 25 34 19 4	1.9 8.7 10.6 24.0 32.7 18.3 3.8	1.9 10.6 21.2 45.2 77.9 96.2 100.0
**Gender**	104		
Female Male	70 34	67.3 32.7	
**Marital status**	103		
Single Married Divorced/Separated Widowed	18 61 7 17	17.5 59.2 6.8 16.5	
**Educational level**	101		
Illiterate 1-4 5-8 9-11 ≥12	2 24 14 24 37	2.0 23.8 13.9 23.8 36.6	2.0 25.7 39.6 63.4 100.0

a Valid percentage;

b Cumulative percentage.

Taking into account all the information gathered by the evaluation team, patients were classified into 10 different primary diagnostic categories, namely Depression, AD, Memory complaints (without objective impairment), MCD, and Anxiety, being the most frequent diagnosis, as shown in Graphic 1.

**Graphic 1:**
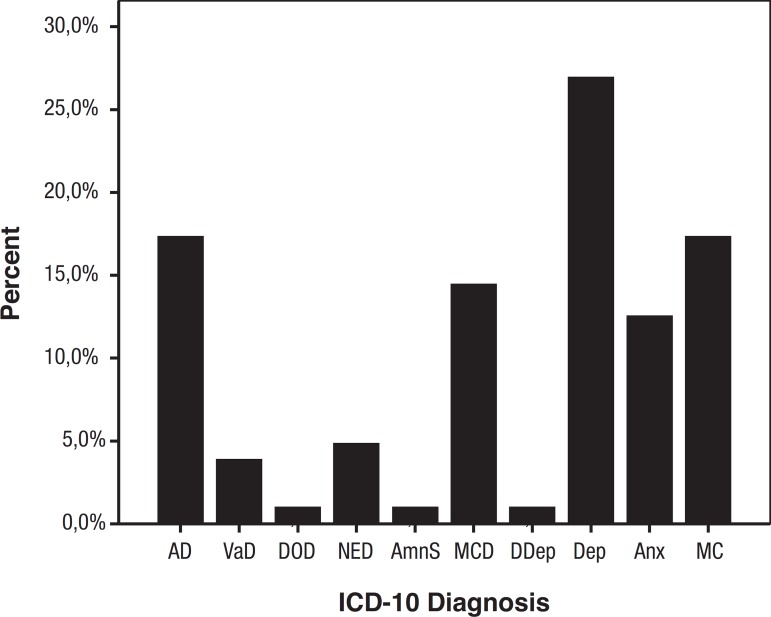
Percentage of etiologic diagnosis, according to ICD-10 (n=104). AD: Alzheimer’s disease; VaD: Vascular dementia; DOD: Dementia secondary to other causes; NED: Non-specified dementia; AmnS: Amnestic syndrome; MCD: Mild cognitive disorder; DDep: Multiple drug dependence; Dep: Depression; Anx: Anxiety; MC: Memory complainers.

To better investigate some characteristics of the most frequent diagnostic groups, we decided to compare patients with dementia, MCD, Depression or Anxiety and Memory complainers, with the frequencies presented in Graphic 2.

**Graphic 2:**
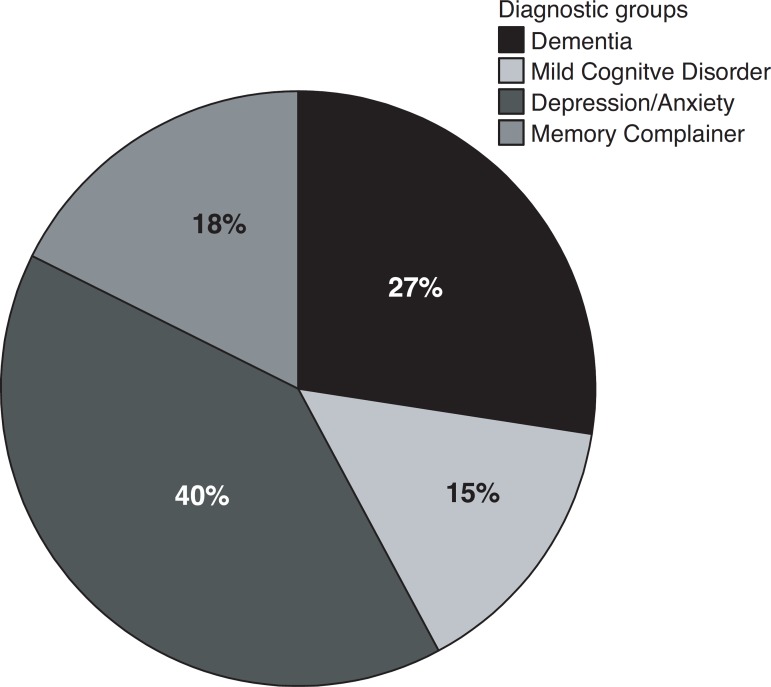
Percentage of diagnosis divided into four groups (n=102).

In order to address the impact of age on diagnosis, we compared the frequencies of the four diagnostic groups by age (20 to 59 years and above 60 years). The results in Table 2 suggest that Dementia was more commonly diagnosed in older subjects, while a higher frequency of Memory complainers was found in the younger group (χ^2^=12.4; p=0.006). There were no differences among the groups regarding gender and marital status.

**Table 2:** Frequency of diagnosis by two age groups.

	Frequency (%) of diagnosis				
Age groups (years)	Dementia	MCD	Dep/Anx	MC	Total
20-59	5 (17.9)	7 (46.7)	21 (51.2)	12 (66.7)	45 (44.1)
>60	23 (82.1)	8 (53.3)	20 (48.8)	6 (33.3)	57 (55.9)

MCD: mild cognitive disorder; Dep/Anx: depression or anxiety; MC: memory complainers.

Table 3 depicts, significant differences regarding group mean ages and schooling, whereby patients with dementia were older than those with depression or anxiety and with memory complaints, and less educated than subjects with memory complaints. Comparing MMSE and CAMCOG mean score, the group with dementia presented significantly lower test scores compared to the other 3 groups, with age and schooling as co-variates.

**Table 3:** Age, schooling, MMSE and CAMCOG scores in 4 diagnostic groups.

	Dementia n=25	MCD n=15	Dep/Anx n=38	MC n=18	Statistical test and p value
Age mean (SD)	67.4 (10.2)^a^	60.1 (14.2)	57.7 (11.3)	52.0 (14.2)	Fb=6.5 p<0.001
Schooling mean (SD)	6.9 (4.6)^c^	10.5 (4.3)	9.7 (4.7)^d^	13.7 (3.1)	Fb=8.6 p<0.001
MMSE mean (SD)	16.6 (5.9)^e^	26.8 (2.0)	27.0 (2.5)	28.8 (0.9)	Ff=42.0 p<0.001
CAMCOG mean (SD)	60.1 (17.4)^g^	80.6 (11.5)	81.3 (10.0)	93.1 (16.6)	Ff=18.2 p<0.001

MCD: mild cognitive disorder; Dep/Anx: depression or anxiety; MC: memory complainers; MMSE: Mini-Mental State Exam; CAMCOG: Cambridge Cognitive Test;

a Age-Dementia vs. Dep/Anx, MC: p<0.01;

^b^ANOVA;

c Schooling-Dementia vs. MC: p<0.001;

d Schooling-Dep/Anx vs. MC: p=0.01;

e MMSE-Dementia vs. MCD, Dep/Anx, MC: p<0.001;

^f^ANCOVA, with age and schooling as co-variates;

g CAMCOG-Dementia vs. MCD, Dep/Anx, MC: p<0.001.

## Discussion

The group of patients evaluated over the first three years of service of the PROTER Memory Clinic was quite heterogeneous, with Depression, AD, Memory complaints (without objective impairment), MCD, and Anxiety being the most frequent diagnoses. As reported earlier, at the Maudsley Hospital Memory Clinic^1^ in the UK, AD and memory complainers were the two most frequently observed diagnoses, with a remarkable absence of potentially reversible conditions. In our sample, not only elderly subjects (45.2% aged less than 60 years) were investigated, where depression or anxiety can be considered potentially reversible conditions because if successfully treated, these patients would present an improvement of cognitive impairment associated to depression and anxiety. This finding reinforced the relevance of a comprehensive evaluation of patients with cognitive complaints, addressing psychiatric conditions that are treatable. The relevance of a systematic psychiatric evaluation of aged subjects referred to a memory clinic was also addressed in Denmark, where 23% of those patients systematically evaluated had a primary psychiatric disease, compared to only 8% of elderly with psychiatric disorders in a reference group at the same institution.^10^

Regarding the memory complainers group, the subjects in our sample were younger and with higher schooling levels, suggesting that they might be concerned about their cognitive performance or might have a higher prevalence of neuropsychiatric disorders in their families. We were not able to investigate these factors in our sample, but female sex, younger age, not being married, being self-referred and having a high prevalence of neuropsychiatric disorders in the family were factors associated with MC diagnosis at the Maudsley Memory Clinic^1^. However, over a 1.9-year average follow-up of subjects classified as cognitively normal in memory disorders clinics,^11^ 36% were re-evaluated, and 65% of them received a diagnosis of dementia or cognitive impairment, suggesting that, even for this group, a periodic re-evaluation might be desirable.

The identification of a sizeable group (around 15%) of subjects classified as MCD is important as, evidence suggests that, carefully selected, many people with MCI, especially Amnestic MCI - are at a high risk of dementia.^12^ We have applied a different diagnostic construct, the MCD from ICD-10, but in fact, many of these subjects would be classified as amnestic or multiple domain mild cognitive impairment, if we were to apply the newly diagnostic criteria suggested for these conditions.^13^ These findings stress the importance of carefully evaluating subjects with memory complaints, as this MCI group might be amenable to prevention or effective pharmacologic and non-pharmacologic treatment in the future.

Considering the frequencies of diagnostic groups by age (20 to 59 years and above 60 years), the rate of dementia was significantly higher in older subjects, while a higher frequency of Memory complainers was found in the younger group. Comparing the diagnostic profile of young (age<60 years) and middle-aged (age>60 years) patients referred to a memory clinic, dementia was also significantly more frequent in the older group, while the cognitive symptoms were rarely diagnosed as dementia but more often reflected other medical and psychiatric disorders.^14^

Considering the MMSE and CAMCOG scores reported in the present study, patients with dementia could be differentiated from the other 3 groups (MCD, Depression or Anxiety and Memory complainers). However, those tests devised for application as a screening instrument (MMSE) or as a brief neuropsychological battery (CAMCOG), were not able to differentiate between subjects with MCD, Depression or Anxiety and Memory Complaints, stressing again the value of a multidisciplinary approach that includes not only a systematic psychiatric evaluation, but also a comprehensive neuropsychological battery applied by a trained neuropsychologist. As cited in a review of clinical and community studies of memory complaints in elderly people, these complaints should be taken seriously, as they might be a possible early sign of dementia.^15^

Despite its limitations, as a preliminary report from an outpatient sample of referred patients with cognitive complaints, the present study showed a higher frequency of psychiatric disorders in this sample, and highlighted the importance of a multi-specialty team to adequately evaluate these subjects. The memory clinic approach should be considered as a model of service to effectively evaluate subjects with cognitive complaints effectively, and to improve the quality of care delivered to this patient group.
